# Sarcopenic Dysphagia: A Narrative Review from Diagnosis to Intervention

**DOI:** 10.3390/nu13114043

**Published:** 2021-11-12

**Authors:** Kuan-Cheng Chen, Ying Jeng, Wei-Ting Wu, Tyng-Guey Wang, Der-Sheng Han, Levent Özçakar, Ke-Vin Chang

**Affiliations:** 1 Department of Physical Medicine and Rehabilitation, National Taiwan University Hospital and National Taiwan University College of Medicine, Taipei 10048, Taiwan; ckc1232001@gmail.com (K.-C.C.); tgw@ntu.edu.tw (T.-G.W.); dshan1121@yahoo.com.tw (D.-S.H.); 2 Department of Physical Medicine and Rehabilitation, Far Eastern Memorial Hospital, New Taipei 22000, Taiwan; 3 Department of Medical Image, National Taiwan University Hospital and National Taiwan University College of Medicine, Taipei 10048, Taiwan; yanijeng@yahoo.com.tw; 4 Department of Physical Medicine and Rehabilitation, National Taiwan University Hospital, Bei-Hu Branch, Taipei 10845, Taiwan; wwtaustin@yahoo.com.tw; 5 Department of Physical and Rehabilitation Medicine, Hacettepe University Medical School, Ankara 06100, Turkey; lozcakar@yahoo.com; 6 Center for Regional Anesthesia and Pain Medicine, Wang-Fang Hospital, Taipei Medical University, Taipei 11600, Taiwan

**Keywords:** aging, sarcopenia, swallowing, assessment, nutrition

## Abstract

Sarcopenia, defined as a decline in muscle mass and function related to aging, affects both limb and swallowing-related muscles. Sarcopenic dysphagia is characterized by decreased swallowing function; therefore, early detection of subclinical dysphagia and subsequent intervention appear to be crucial in the elderly. Numerous tools have been employed to measure the function, strength, and mass of swallowing-related muscles in sarcopenic elderly. The swallowing function can be evaluated by questionnaires like Eating Assessment Tool, Functional Oral Intake Scale, and Food Intake Level Scale, and tests such as the modified water swallowing test and videofluoroscopic swallowing study. Surface electromyography and high-resolution manometry can be applied for quantifying swallowing-related muscle strength. Modalities such as ultrasonography and magnetic resonance imaging are capable of estimating the swallowing muscle mass. In patients with sarcopenic dysphagia, a thorough assessment should be given followed by an integrated intervention combining swallowing muscle strengthening, nutrition support, food texture modification, physical, and occupational therapies. This article aimed to comprehensively summarize the diagnostic criteria/tools as well as their associations/performance in sarcopenic dysphagia. The intervention strategy will also be narrated in this review.

## 1. Introduction

Sarcopenia is described as a decline in muscle mass, strength, and physical function. The pathophysiology of sarcopenia comprises primary and secondary causes. The former is considered as age-related changes without other specific pathologies. Sarcopenia derived from a systemic disease (malnutrition, malabsorption, physical inactivity, advanced organ failure, inflammatory/endocrine disease, or malignancy) is termed secondary sarcopenia [1]. Several adverse health outcomes were found in the elderly with sarcopenia, such as increased mortality and longer hospitalization [2]. Furthermore, like the extremity muscles, swallowing-related muscles can also be affected in these patients [3]. Previous studies evaluating the elderly demonstrated that sarcopenia was an independent risk factor for dysphagia [4,5]. Although there are no golden diagnostic criteria for sarcopenic dysphagia, many tools have been used for the evaluation. The diagnosis of sarcopenic dysphagia heavily depends on the assessment of swallowing function whereby comprehensive evaluation and prompt intervention are paramount. Since sarcopenic dysphagia has become an important issue concerning the nutritional status of the elderly, the present narrative review aimed to explore the current evidence in the pertinent literature.

## 2. Definition

### 2.1. Sarcopenia

Sarcopenia is a clinical syndrome that refers to a gradual and generalized loss of skeletal muscle mass and strength [2]. The decline of muscle mass is about 3–8% per decade after the age of 30 years and rises to 15% after 70 years of age [6,7]. Recently, the concept of the gut-muscle axis has been proposed to play a crucial role in the development of sarcopenia. The gut microbiota has been found to impact muscle volume and performance through mediating inflammatory reactions, immunity, endocrine function, and energy metabolism [8,9]. According to the European Working Group on Sarcopenia in Older People (EWGSOP) [1] and Asian Working Group for Sarcopenia (AWGS) [10] diagnostic criteria, sarcopenia is defined as low muscle mass, strength, and/or physical performance. The cut-point value of sarcopenia revised by the European Working Group on Sarcopenia in Older People (EWGSOP) in 2018 is (1) <7.0 kg/m^2^ in men and <5.5 kg/m^2^ in women for low muscle quantity; (2) handgrip strength < 27 kg for men and < 16 kg for women for low muscle strength; (3) 6-m walk < 0.8 m/s, Short Physical Performance Battery score ≤ 8, 5-time chair stand test ≥ 15 s, timed up-and-go test ≥ 20 s, or 400-m walk ≥ 6 min for low physical performance [11]. Moreover, the cut-point value suggested by Asian Working Group for Sarcopenia (AWGS) 2019 consensus is: (1) <7.0 kg/m^2^ in men and <5.4 kg/m^2^ in women on dual-energy X-ray absorptiometry; and <7.0 kg/m^2^ in men and <5.7 kg/m^2^ in women on bioimpedance analysis; (2) handgrip strength < 28 kg for men and < 18 kg for women for low muscle strength; (3) 6-m walk < 1.0 m/s, Short Physical Performance Battery score ≤ 9, or 5-time chair stand test ≥ 12 s for low physical performance [12]. Due to the variations of its diagnostic criteria, the prevalence of sarcopenia ranges between 1% and 29% in the elderly [13]. Of note, sarcopenia may also reduce the strength of swallowing muscles, leading to dysphagia [14,15].

### 2.2. Dysphagia

Dysphagia is defined as difficulty in eating and swallowing [16], presenting with impaired or prolonged transit of food or liquids from the oral cavity to the esophagus [17]. It commonly ensues as a consequence of diseases like esophageal cancer, stroke, and Parkinson’s disease [18]. The swallowing process can be divided into the following four stages: the oral preparatory, oral, pharyngeal, and esophageal phases. The dysfunction in any of the aforementioned stages can cause dysphagia [19]. The association of dysphagia with several adverse health outcomes—e.g., malnutrition, dehydration, respiratory infections, aspiration pneumonia, increased readmissions, institutionalization, and mortality-have been identified [17]. As such, early detection of subclinical dysphagia and subsequent intervention are crucial in the elderly.

### 2.3. Sarcopenic Dysphagia

Dysphagia caused by sarcopenia is categorized as sarcopenic dysphagia [3]. Indisputably, dysphagia is also a risk factor for malnutrition in older patients. The mechanism behind sarcopenic dysphagia is thought to be a decline in the swallowing-related muscle mass and strength. Age-related loss of swallowing muscle mass can be manifested as a decrease in the thickness of the tongue [20], geniohyoid muscle [21], and pharyngeal wall, and an increase in the pharyngeal lumen size [22]. These changes contribute to decreased tongue strength, reduced range of tongue motion, weakened pharyngeal muscle contraction, and deteriorated endurance of swallowing muscles, all of which are the risk factors of dysphagia [23].

**Figure 1:**
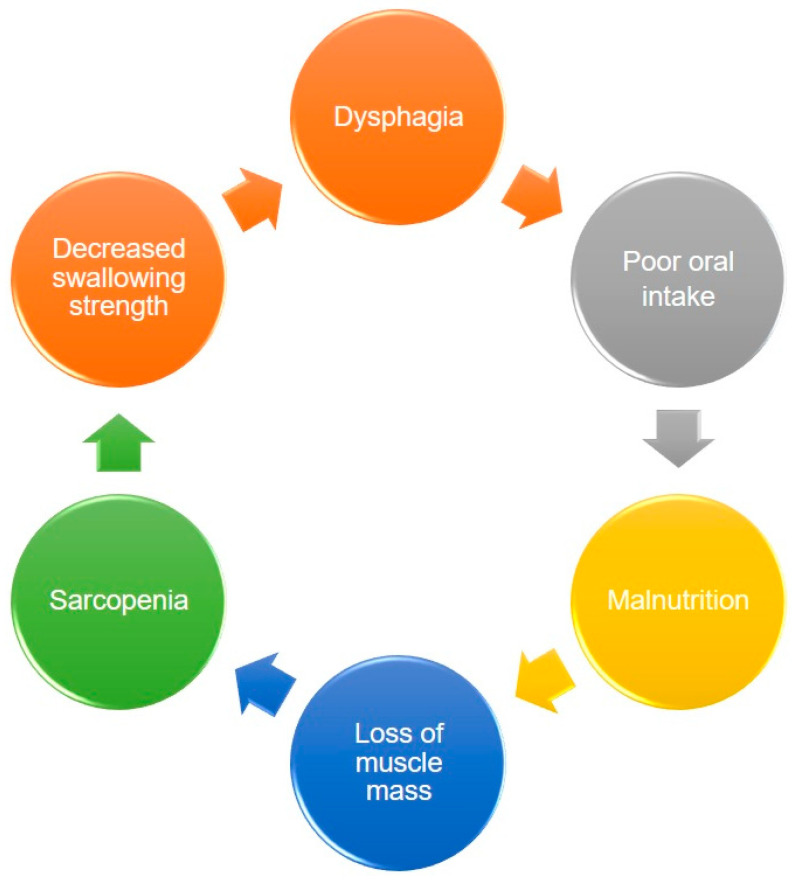
The vicious circle between dysphagia, malnutrition, and sarcopenia.

Dysphagia increases the risk of malnutrition due to reduced oral intake. Patients who can achieve full oral intake without additional nutrition support through parenteral routes are able to obtain higher energy contents from food than those who cannot [24]. Decreased nutrition support also leads to weight loss and disrupted synthesis of skeletal muscles, which consequently result in further development of sarcopenia. Therefore, a vicious cycle of sarcopenia and dysphagia eventually becomes inevitable (Figure 1).

The consensus on the diagnosis of sarcopenic dysphagia has been established at the 19th Annual Meeting of the Japanese Society of Dysphagia Rehabilitation [23]. The coexistence of dysphagia and sarcopenia is mandatory for the diagnosis. In other words, if the main cause of dysphagia is sarcopenia accompanied by loss of swallowing muscle mass identified on imaging modalities, ‘definite sarcopenic dysphagia’ is confirmed. If sarcopenia could not be ruled out as a cause of dysphagia, ‘probable sarcopenic dysphagia’ is considered. If the main cause of dysphagia is sarcopenia with the coexistence of diseases that may be linked to dysphagia (such as stroke or head/neck cancer), ‘possible sarcopenic dysphagia’ is defined (Table 1) [25].

**Figure 2:**
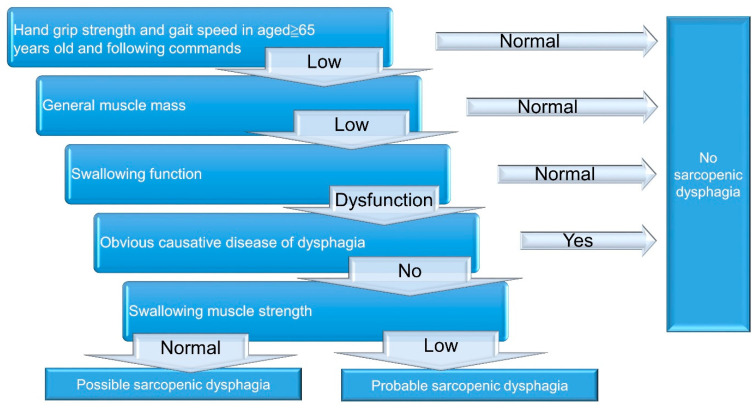
A 5-step diagnostic algorithm for the diagnosis of sarcopenic dysphagia.

A 5-step diagnostic algorithm has been developed by the Working Group on Sarcopenic Dysphagia [23] (Figure 2). It categorizes the examinees into three groups: probable sarcopenic dysphagia, possible sarcopenic dysphagia, and no sarcopenic dysphagia. Patients with diseases (other than sarcopenia) directly leading to dysphagia are excluded. Compared with the aforementioned initial consensus, the advantage of this diagnostic algorithm is the use of tongue pressure to represent its strength without assessing swallowing muscle mass. The cut-off value for defining low tongue pressure is 20 kPa. In 2017, Mori et al. [25] enrolled 119 old inpatients for verifying the reliability and validity of the 5-step algorithm. The intra-class coefficients for intra- and inter-rater reliability were 0.87 (95% confidence interval [CI]: 0.73–1.01), and 0.98 (95% CI: 0.92–1.02), respectively. In 2019, Wakabayashi et al. [26] recruited 108 patients to assess the prevalence and prognosis of sarcopenic dysphagia by using the same algorithm. The study revealed the prevalence of sarcopenic dysphagia as 32% in patients who require rehabilitation for swallowing dysfunction. The authors also identified that the swallowing function at discharge was worse in patients with sarcopenic dysphagia vs. non-sarcopenic dysphagia.

## 3. Tools for the Evaluation of Sarcopenic Dysphagia

### 3.1. Overview

There are several validated clinical and instrumental methods to evaluate dysphagia in sarcopenic individuals. The tools used in the evaluation of sarcopenia include muscle mass, muscle strength, and physical performance. Dual-energy X-ray absorptiometry (DXA) and bioimpedance analysis (BIA) have been commonly applied for the measurement of muscle mass. The dynamometer is used to evaluate grip strength. The six-minute walk test (6MWT), Short Physical Performance Battery score (SPPB), five-time chair stand test (5TSTS), timed up-and-go test (TUG), and 400 m walk test (400MWT) are employed to assess the physical performance. Similar to the assessment of sarcopenia, the muscle volume and strength—as well as function related to swallowing—should be appraised in participants suspected of sarcopenic dysphagia. Questionnaires, swallowing tests, and videofluoroscopic swallowing study (VFSS) can be utilized to determine the swallowing function. Measurement of tongue pressure, lip force, jaw-opening force, surface electromyography (sEMG), and high-resolution manometry (HRM) can be employed for quantifying swallowing-related muscle strength. Ultrasonography and magnetic resonance imaging (MRI) are modalities useful for estimating the swallowing muscle mass (Table 2). The cut-off points for the tools for the diagnosis of sarcopenic dysphagia from the available literature are presented in Table 3. As the evaluation tools for sarcopenia have been well addressed by existing literature, the prevent review would focus on the assessment of the swallowing component.

### 3.2. Swallowing Function Evaluation: Questionnaires & Swallowing Tests

The Eating Assessment Tool (EAT-10) is a concise questionnaire that can be applied to screen dysphagia [27]. It consists of 10 items and each item is scored from 0 (no problem) to 4 (severe problem). A total score of 3 or more is defined as abnormal swallowing function [27]. In a survey of 235 individuals with voice and swallowing disorders, Belafsky et al. [27] reported the internal consistency to be 0.960 in Cronbach alpha and the test-retest reliability (expressed by intra-class correlation coefficients) to range from 0.72 to 0.91. In 2017, Wakabayashi et al. [28] screened 83 cancer patients using EAT-10 and demonstrated a higher prevalence of dysphagia in the sarcopenia group than in the non-sarcopenia group. In 2021, Ozer et al. [29] investigated 512 patients (aged 60 years and older) in a geriatric outpatient clinic of a tertiary center. They reported that EAT-10 was positively correlated with age, geriatric depression scale, and the timed up-and-go score; but negatively correlated with mini-nutritional assessment-short form, mini-mental state examination, handgrip strength, and hemoglobin values.

Dysphagia severity scale (DSS) and the repetitive saliva swallowing test (RSST) are common means to assess dysphagia. DSS is a 7-point ordinal scale with a lower score indicating a worse condition [30]. DSS had high interrater (90%) and intrarater (93%) agreements, established by four clinicians on 135 patients in a teaching hospital [31]. RSST examines the ability to voluntarily swallow repeatedly by asking the participant to swallow saliva as many times as possible in 30 s [32]. Three or more dry swallows within 30 s is considered normal. In a study examining 120 healthy adults and 40 stroke patients in a geriatric sub-acute stroke unit, the sensitivity and specificity of RSST for screening dysphagia were found to be 69% and 93%, respectively [33]. Regarding the association of DSS and RSST with sarcopenic dysphagia, Shiozu et al. [15] enrolled 77 old adults in a geriatric health service facility and reported decreased scores of DSS, RSST, Functional Independence Measure, and Mini Nutritional Assessment-Short Form in the sarcopenia group.

Functional Oral Intake Scale (FOIS) is an observer-rated ordinal scale focusing on oral intake, ranging from 1 (worst) to 7 (normal). Patients with an FOIS ≤ 5 are considered to have dysphagia [34]. In 2016, Maeda et al. [4] enrolled 95 older hospitalized patients and reported decreased skeletal muscle index, Barthel Index, Mini Nutritional Assessment Short Form, energy intake at seven days after admission, and Mini-Mental State Examination score in the group with a FOIS level ≤ 5. In 2020, Nagano et al. [35] assessed 89 older female patients with hip fractures and found that all patients with post-operative dysphagia (diagnosed by FIOS) had preoperative sarcopenia. In 2016, Maeda et al. [36] included 224 older adults in an acute ward and found that the group with an FOIS ≤ 5 had lower values of Barthel Index, body mass index, Mini-Nutritional Assessment-short Form, and skeletal muscle index when compared with the group without dysphagia. In 2020, Silva et al. [37] investigated 71 men with head/neck cancer and reported that the dysphagia group with an FOIS ≤ 5 had lower body mass index, skeletal muscle mass and handgrip strength, and a higher prevalence of sarcopenia than the non-dysphagia group.

Food Intake Level Scale (FILS) is a 10-point scale to determine the severity of swallowing dysfunction. Levels 1–3, 4–6, and 7–10 indicate various degrees of non-oral feeding, oral food intake with alternative nutrition, and oral food intake alone, respectively [38]. The interrater and intra-rater reliability (quantified by kappa coefficients) ranged from 0.70 to 0.90 and 0.83 to 0.90, respectively—from the data of three clinicians assessing 30 inpatients at a rehabilitation department of a general hospital [38]. In 2015, Wakabayashi et al. [39] studied 111 cancer patients with dysphagia and reported that the group of non-oral feeding (FILS levels 1–3) had lower skeletal muscle mass, Barthel index and hemoglobin, and a longer period of hospitalization. In 2017, Wakabayashi et al. [40] found that a lower FILS score at discharge after cardiovascular surgery was associated with post-operative skeletal muscle mass loss. In 2018, Yoshimura et al. [41] surveyed 637 patients in a rehabilitation ward and showed that FILS scores were positively correlated with skeletal muscle mass index, Mini Nutritional Assessment-short form, and handgrip strength.

The modified water swallowing test (MWST) is a simple measurement to detect aspiration by swallowing water [42]. A total of 3 mL cold water is poured over the floor of the mouth by using a 5 mL syringe, and then the patients are instructed to swallow [43]. Inability to swallow with choking/breathing changes, swallowing with changes in breathing patterns, or swallowing with choking/wet voices are regarded as dysphagia. The sensitivity and specificity of MWST for detecting aspiration are 70% and 88%, respectively [43,44]. The MWST requires a combination of cervical auscultation to check breathing sounds before and after drinking the water [45,46]. Any signs of choking, effortful breathing, or wheezing by cervical auscultation after swallowing are viewed as possible dysphagia [47]. In 2017, Sagawa et al. [48] examined 310 Japanese elderly at a daycare center using MWST and cervical auscultation. They observed that the skeletal muscle mass of men in the dysphagia group was lower than in the non-dysphagia group.

### 3.3. Videofluoroscopic Swallow Study

**Figure 3:**
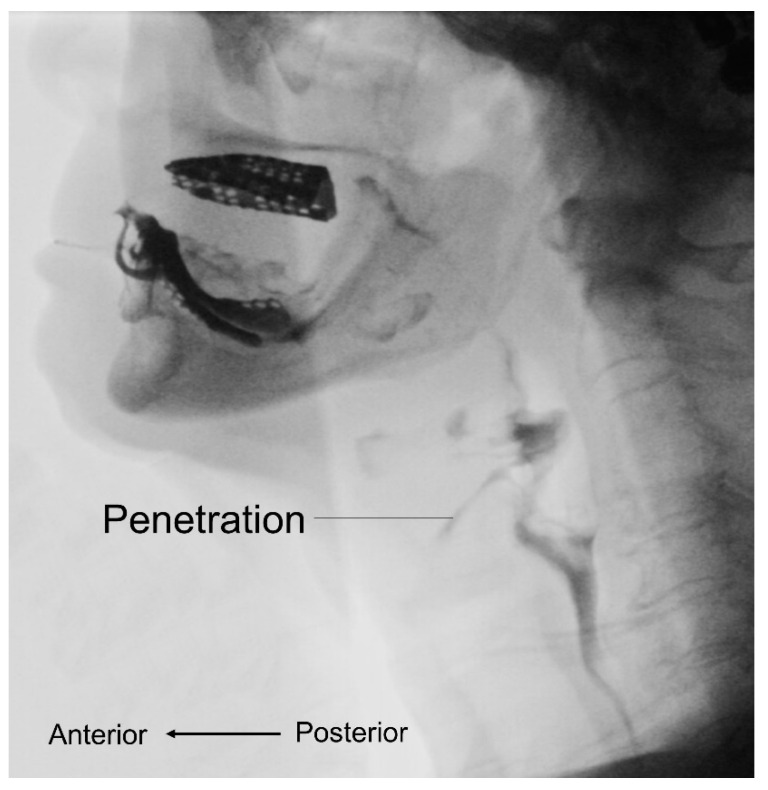
Videofluoroscopic swallow study shows abnormalities during the bolus transport such as penetration.

The use of videofluoroscopic swallow study (VFSS) provides objective information on bolus transport during swallowing (Figure 3). Two case reports demonstrated the VFSS finding in patients with sarcopenic dysphagia to include residue in the valleculae and pyriform sinuses, aspiration [49], and reduced maximal superior and anterior displacement of the hyoid bone and thyroid cartilage during swallowing [50]. In 2019, Miyashita et al. [51] enrolled 132 patients with swallowing complaints and showed that sarcopenic male participants had lower laryngeal upward movements during swallowing and wider pharyngeal areas than the controls. In women, only an enlarged pharyngeal area was identified in the sarcopenic group in contrast to the non-sarcopenic group.

### 3.4. Maximal Isometric Tongue Pressure

**Figure 4:**
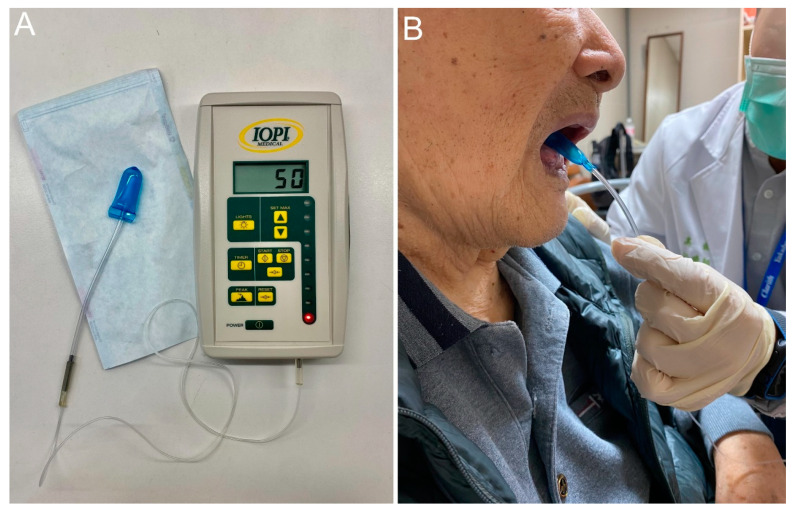
The Iowa oral performance instrument (**A**) and how it is used for obtaining maximal tongue pressure (**B**).

Maximal isometric tongue pressure (MTP) is measured by a device with a disposable oral balloon probe and a plastic pipe, such as JMS measurement apparatus (JMS, Hiroshima, Japan) or Iowa Oral Performance Instrument (IOPI). The balloon is placed in the mouth and the plastic pipe is bitten at the middle of the central incisors with the lips closed. Patients are then asked to raise their tongue and press the balloon to the hard palate for several seconds with the full effort to obtain the maximal tongue pressure (Figure 4). In 2015, Maeda et al. [52] enrolled 104 patients without stroke or neurodegenerative disease and revealed that the MTP was positively correlated with Barthel index, serum albumin concentration, body mass index, Mini Nutritional Assessment-short form, and arm muscle cross-sectional area. In 2017, Sakai et al. [53] enrolled 174 adult inpatients in a rehabilitation ward and reported that MTP was positively associated with Barthel index, Mini Nutritional Assessment-short form, albumin, body mass index, grasp strength, and FOIS. In 2018, Kaji et al. [54] enrolled 144 patients with diabetes mellitus and found that MTP was positively correlated with skeletal muscle mass and handgrip strength. In 2018, Suzuki et al. [55] enrolled 245 community-dwelling older women and identified that decreased MTP and oral diadochokinesis were found in the sarcopenia and dynapenia groups. In 2019, Kobuchi et al. [56] assessed 54 elderly residents in a nursing home and found that MTP was positively correlated with grip strength, skeletal muscle mass, and cross-sectional area of the geniohyoid muscle. Furthermore, the association between decreased MTP and malnutrition in community-dwelling elder subjects was identified by Chang et al. [57] in 2021. A recent meta-analysis incorporating ten studies also confirmed the significant association between declined MTP and sarcopenia [58].

### 3.5. Jaw-Opening Force

Jaw-opening force can be employed to measure the strength of the suprahyoid muscles by using the device like jaw-opening force trainer KT2016 (Livet Inc., Tokyo, Japan), a spherometer with a head encircling belt, two belts to secure the mandible to the head-encircling belt, a chin cap, and a dynamometer. Participants are asked to open their jaws with their full effort to obtain the value of maximum force. In 2017, Machida et al. [59] enrolled 197 community-dwelling older adults to examine MTP and jaw-opening force in participants with or without sarcopenia. They observed that sarcopenia was an independent factor affecting tongue pressure in male and female subjects. However, the jaw-opening force was only associated with sarcopenia in the male group.

### 3.6. Lip Force

Lip force can be measured by using a device such as Lip de Cum (Cosmo Instruments Co., Ltd., Tokyo, Japan). This instrument records the combinational forces on the vertical axis from sensors implanted into the upper and lower plastic lip holder. The participants are asked to close the lips with the maximal effort to obtain the highest value of lip force. In 2019, Sakai et al. [60] investigated 245 patients admitted for post-acute care and they reported that sarcopenic dysphagia was inversely associated with lip pressure in male and female subjects. The area under curve to discriminate sarcopenic dysphagia was 0.88 (95% CI, 0.81–0.95) for men and 0.84 (95% CI, 0.77–0.90) for women. Furthermore, the cut-off values of lip force for sarcopenic dysphagia were 10.4 Newton for men and 8.5 Newton for women.

### 3.7. Surface Electromyography

**Figure 5:**
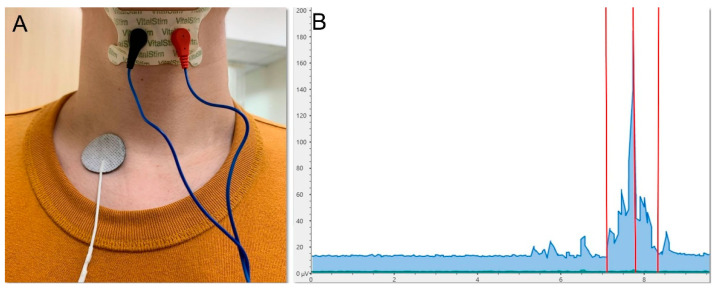
Electrode placement (**A**) and signal display (**B**) for the electromyographic assessment of swallowing muscle activity. Left red vertical line, onset of swallowing; middle red vertical line, peak amplitude; right red vertical line, end of swallowing.

Surface electromyography (sEMG) can be applied to evaluate swallowing muscle activity (Figure 5). The electrodes are put on the skin near the lip over the submental area or suprahyoid region to detect the muscle activity of the orbicularis oris superior and inferior, masseter, submental muscle groups (anterior belly of the digastric, mylohyoid, and geniohyoid), and the laryngeal strap (infrahyoid and thyrohyoid) muscles. These muscles are selected because they are superficial and involved in the oral and pharyngeal phases of swallowing. In 2004, Vaiman et al. [61] assessed swallowing physiology in 300 adults in Israel by using sEMG and found that the swallowing duration was positively correlated with the increase in age. In 2020, Sakai et al. [62] investigated 60 old inpatients in a Japanese rehabilitation hospital. They found that patients with sarcopenic dysphagia had longer durations and higher amplitudes of submental sEMG activity in the suprahyoid muscles during swallowing compared with the non-dysphagic group.

### 3.8. High-Resolution Manometry

High-resolution manometry has been employed to assess pharyngeal dysphagia by the measurement of intra-lumen pressure of the gastrointestinal tract [63]. During swallowing, the upper esophageal sphincter pressure decreases for the passage of the food bolus. Increased upper esophageal sphincter pressure would cause impaired food bolus transit, leading to residue in the pyriform sinuses and post-swallowing aspiration. In 2021, Kunieda et al. [64] assessed 16 Japanese elderly patients with pharyngeal dysphagia in a rehabilitation hospital. They reported that patients with sarcopenic dysphagia had weaker pharyngeal contraction force and higher upper esophageal sphincter pressure. Regarding normal swallowing physiology, an elevation in upper esophageal sphincter pressure can be compensated by the increase in pharyngeal contraction [65]. However, the compensatory mechanism may be compromised in patients with sarcopenic dysphagia i.e., causing a decrease in pharyngeal contractility.

### 3.9. Ultrasonography

**Figure 6:**
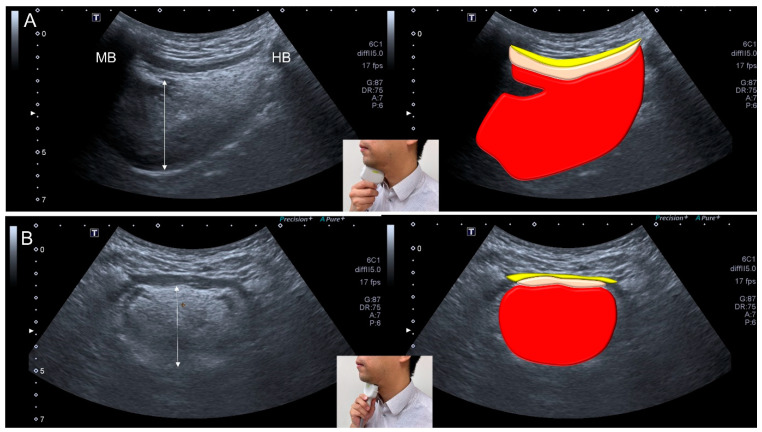
Ultrasonographic images and schematic drawings of tongue and adjacent muscles in the sagittal (**A**) and coronal (**B**) planes. Tongue muscle: red color block; geniohyoid muscle, pink color block; mylohyoid muscle, yellow color block; MB: mandible; HB: hyoid bone; double arrowed line, thickness of the tongue muscle.

Ultrasonography has benefits such as noninvasiveness, non-radiation exposure, and the capability of dynamic assessment. Moreover, tongue and geniohyoid muscles can be easily/promptly visualized by ultrasonography [14,20,66] (Figure 6). In 2012, Tamura et al. [20] found that tongue thickness (measured by ultrasonography) was positively correlated with triceps skinfold thickness and cross-sectional area (CSA) of the arm, and negatively correlated with body weight in 104 old adults. In 2018, Ogawa et al. [14] identified decreased CSA and increased echogenicity of the tongue muscles in patients with sarcopenic dysphagia. The sensitivity and specificity of the tongue CSA for discriminating sarcopenic dysphagia were 0.389 and 0.947, respectively. The sensitivity and specificity of the geniohyoid muscle echogenicity for discriminating sarcopenic dysphagia were 0.806 and 0.632, respectively. In 2019, Kobuchi et al. [56] assessed 54 elderly persons from nursing homes and found that CSA of the geniohyoid muscle was positively correlated with skeletal muscle mass index and tongue pressure. Decreased geniohyoid muscle CSA and tongue pressure were shown to be associated with sarcopenia. In 2021, Mori et al. [67] enrolled 36 inpatients with sarcopenic dysphagia and reported that geniohyoid muscle CSA was positively correlated with MTP and CSA of tongue muscle.

**Figure 7:**
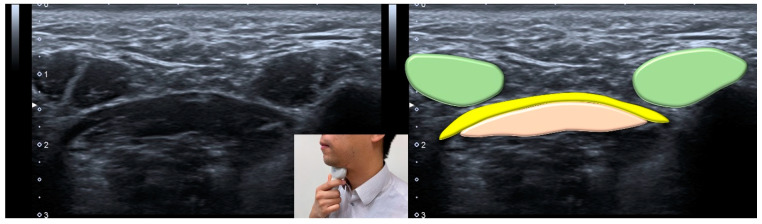
Ultrasonographic images and schematic drawing of the anterior belly of the digastric muscle in the coronal plane. Digastric muscle, green color block; mylohyoid muscle, yellow color block; geniohyoid muscle, pink color block.

Ultrasonography can also be applied for the evaluation of the digastric muscle (Figure 7). In 2020, Ogawa et al. [68] investigated 45 elderly patients and reported smaller digastric muscle mass in patients with sarcopenic dysphagia vs. controls. The digastric muscle mass was identified as an independent factor for predicting sarcopenic dysphagia, with a sensitivity of 0.692 and a specificity of 0.737.

**Figure 8:**
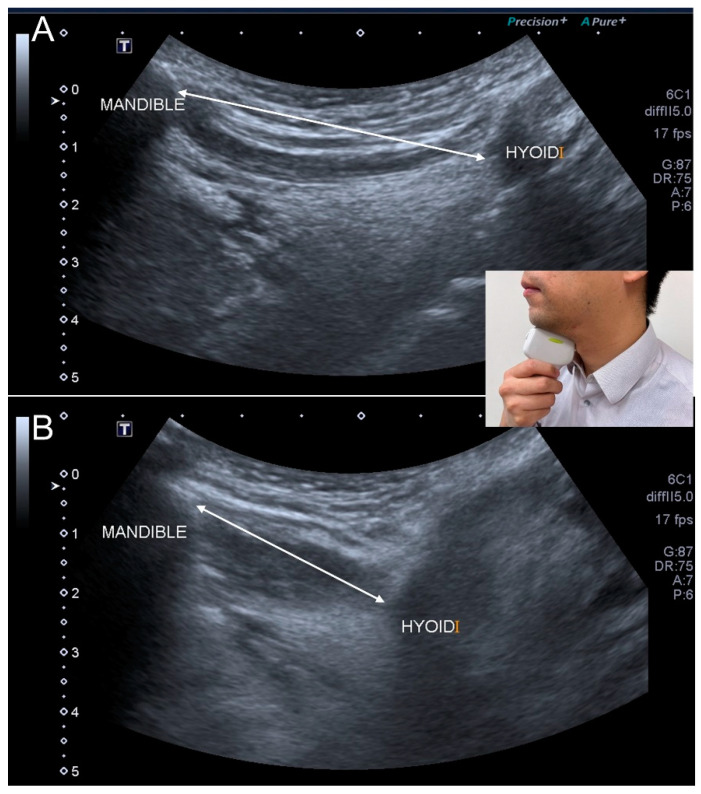
Ultrasonographic images show the hyoid movement at the onset of swallowing (**A**) and the moment of maximal displacement (**B**). Double-headed line, the distance between the mandible and hyoid bone.

Hyoid movement is related to the opening of the upper esophageal sphincter. Individuals are asked to swallow 3 mL of water during which the hyoid displacement is measured by submental ultrasonography (Figure 8). In 2020, Chen et al. [69] enrolled 94 community-dwelling residents and reported increased maximal hyoid displacement and motion velocity in patients with sarcopenia. The authors considered the condition to be a compensatory response for swallowing muscle weakness.

### 3.10. Magnetic Resonance Imaging

**Figure 9:**
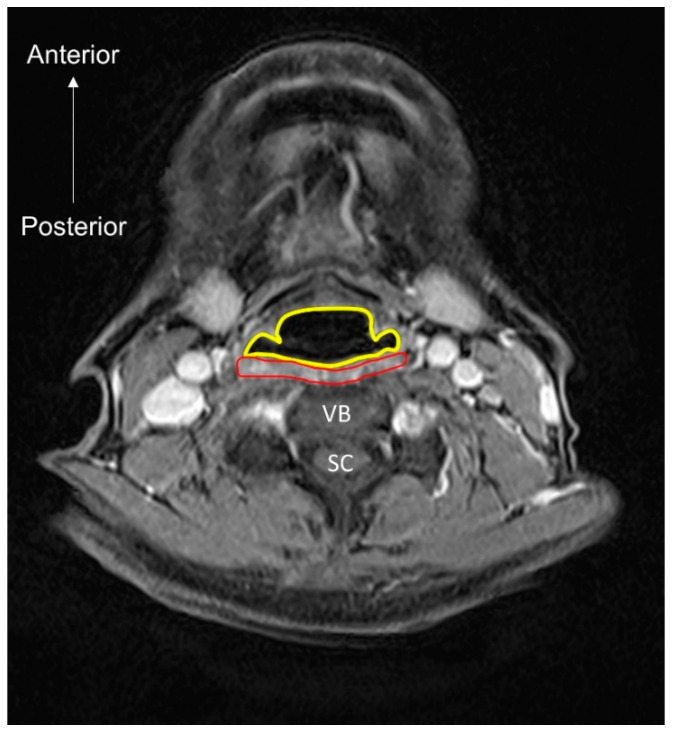
MRI (axial view) of the pharyngeal lumen (encircled by the yellow line) and muscle (encircled by the red line). VB, vertebral body; SC, spinal cord.

Magnetic resonance imaging (MRI) is a useful instrument to assess swallowing-related muscle mass and adjacent structures (Figure 9). In 2015, Molfenter et al. [22] reviewed neck MRI of 60 older women without dysphagia and reported decreased wall thickness and enlarged lumen of the pharynx. In 2021, Sakai et al. [70] assessed 70 Japanese patients with acute stroke (a common cause of secondary sarcopenia) and revealed decreased thickness of the temporalis muscle in parallel with an increase in dysphagia. In 2021, Nakao et al. [71] enrolled 20 old and 20 young adults whose MRI showed that the intramuscular fatty infiltration of tongue, pharyngeal, and geniohyoid muscles increased and the genohyoid muscle mass decreased with aging. They also found a positive correlation between MTP and swallowing-related muscle mass. In 2021, another MRI study conducted by Nakao et al. [72] demonstrated that MTP was negatively correlated with tongue fat mass and percentage.

## 4. Intervention

### 4.1. Overview

**Figure 10:**
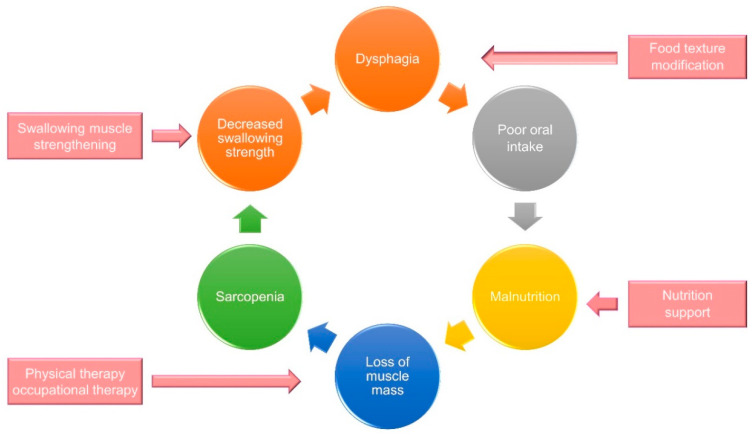
A multidisciplinary intervention strategy to break the vicious circle between sarcopenic dysphagia and malnutrition.

**Figure 11:**
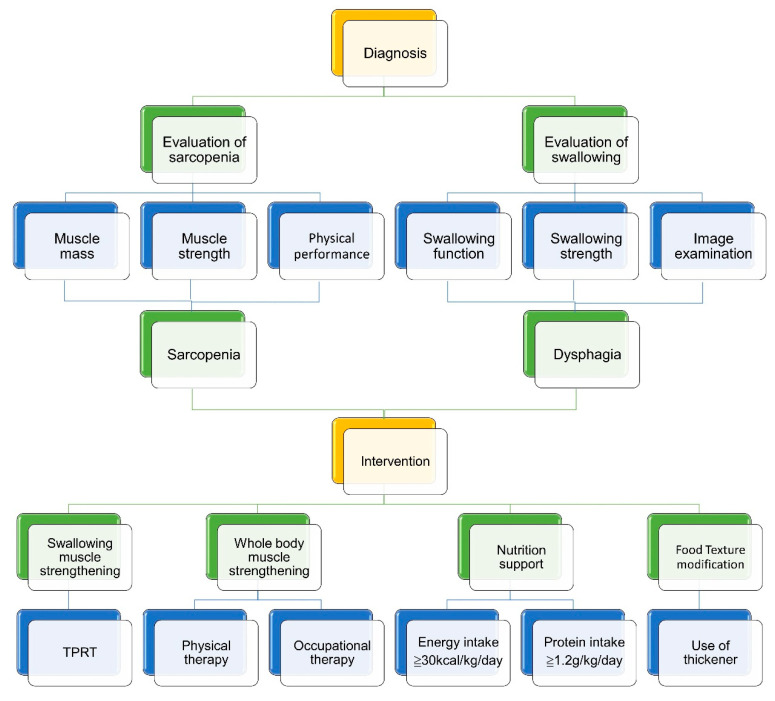
An integrated protocol for managing patients with sarcopenic dysphagia. TPRT, tongue-pressure resistance training.

Care for sarcopenic dysphagia requires a multi-disciplinary strategy. The interventions to break the vicious circle between dysphagia and malnutrition include swallowing muscle strengthening, physical therapy, occupational therapy, nutrition support, and texture modification of food (Figure 10). Cooperation with the patients’ families/caregivers is paramount for the treatment success [73]. Below is an integrated protocol for managing patients with sarcopenic dysphagia [73,74,75] (Figure 11).

### 4.2. Swallowing Muscle Strengthening

Swallowing muscle strengthening-including lingual resistance exercises, breathing training, and tongue exercises can be applied in patients with sarcopenic dysphagia [49]. Tongue-pressure resistance training (TPRT) is the most commonly used strengthening exercise. To perform TPRT, the patient begins to push his/her entire tongue against the palate as hard as possible for 10 s with the mouth closed and then rests for 10 s. In 2017, Kim et al. [76] suggested performing TPRT five times, two sets per day for a month in subacute stroke survivors with dysphagia.

In 2015, Robbins et al. [77] applied a similar resistance exercise using IOPI for 8 weeks in 10 old healthy adults. The participants were asked to compress an air-filled bulb between the tongue and hard palate 30 times, three sets per day with 60–80% of one- repetition maximum. Following training, the peak swallowing pressures increased significantly and the lingual muscle volume (on MRI) improved by an average of 5.1%. In 2019, Namiki et al. [78] enrolled 18 patients having presbyphagia with symptoms such as coughing and choking. They reported that anterior/superior hyoid movements, tongue pressure, and width of the upper esophageal sphincter improved after TPRT.

### 4.3. Nutrition Support

Poor nutritional status is a core characteristic of sarcopenic dysphagia whereas intensive nutrition therapy [79] combined with muscle strength [80] is helpful in increasing the muscle mass. Nutritional improvement is associated with the improvement of dysphagia in patients with pneumonia and malnutrition [81]. In 2020, Nagano et al. [82] found that an energy intake of ≥30 kcal/kg/day and a protein intake of ≥1.2 g/kg/day significantly improved tongue strength. In 2021, Shimizu et al. [83] enrolled 110 patients with sarcopenic dysphagia and studied the benefits of high-energy supplements on swallowing function. FILS and Functional Independence Measure at discharge were significantly higher in the group given food of ≥30 kcal/ideal body weight/day (kg) than the group given food of less energy.

Dysphagia can cause malnutrition due to poor oral intake. Moreover, malnutrition is a risk factor for the development of secondary sarcopenia and sarcopenic dysphagia. In 2019, Tanıgör et al. [84] evaluated the prevalence of nutritional deficits and status of dysphagia and sarcopenia in 128 patients. Scores of EAT-10 and Mini Nutritional Assessment were found to be worse in sarcopenic individuals than non-sarcopenic controls.

### 4.4. Physical and Occupational Therapy

In addition to swallowing muscle training, intervention for the declined whole-body muscle mass and strength should be given in patients with sarcopenic dysphagia. A combination of exercise and nutritional interventions is essential for the elderly with sarcopenia. Physical therapy emphasizing the strengthening of four limbs brings collateral benefits for swallowing-related muscles [49,85]. In 2020, Nagano et al. [82] enrolled 95 sarcopenic patients to investigate the impact of 2-month physical and occupational therapy with nutritional support on swallowing function. They reported an increase in MTP after intervention without additional swallowing training. Moreover, they found that a higher amount of energy and protein intake were positively correlated with an improvement in MTP.

### 4.5. Texture Modification of Food

Texture modification of food should be incorporated to improve the safety and efficiency of oral eating in patients with sarcopenic dysphagia. In 2016, Carrion et al. [86] assessed 133 older patients with oropharyngeal dysphagia by using VFSS to explore the association between nutritional status and swallowing. Only 38.2% of the patients with poor nutrition status (defined by Mini-Nutritional Assessment ≤ 23.5) could swallow liquid safely. Furthermore, 61.7% of the patients required a thickener of nectar viscosity and 25.5% required a thickener of spoon-thick. This finding suggested that texture modification of food would be needed for patients with sarcopenic dysphagia to ensure swallowing safety.

## 5. Future Perspectives

Through our literature search, we identified that the diagnostic criteria varied across different studies, which would lead to variations of the prevalence of sarcopenic dysphagia. A systematic review and meta-analysis should be implemented in the future to obtain the summarized estimate of the prevalence in general or disease-specific populations. Furthermore, compared with the diagnostic or observational studies for sarcopenic dysphagia, the number of intervention trials is largely insufficient. The long-term benefit of treatments for sarcopenic dysphagia remains unclear. Therefore, more randomized controlled trials implementing the multidisciplinary therapeutic approach with an extended duration of follow-up are desperately desired henceforth.

## 6. Conclusions

The coexistence of dysphagia and sarcopenia is the key element for the diagnosis of sarcopenic dysphagia. Several tools are useful for the evaluation of different swallowing components in sarcopenic individuals. These encompass questionnaires and swallowing tests, VFSS, instruments measuring tongue/lip/jaw-opening strength, sEMG, HRM, ultrasonography, and MRI. A multidisciplinary approach combing swallowing muscle strengthening, nutrition support, physical/occupational therapy, and texture modification of food should be given to patients with sarcopenic dysphagia once a diagnosis is confirmed. The goal of these interventions is to recover whole-body and swallowing-related muscle mass and function, breaking the vicious circle between dysphagia and malnutrition.

## Figures and Tables

**Table 1:** Diagnostic criteria for sarcopenic dysphagia.

Item	Criteria
1	Presence of dysphagia
2	Presence of whole-body sarcopenia
3	The results of imaging tests (computed tomography, magnetic resonance imaging, ultrasonography) are consistent with loss of swallowing muscle mass
4	The causes of dysphagia are excluded except for sarcopenia
5	The main cause of dysphagia is considered to be sarcopenia

Definite diagnosis: 1, 2, 3, 4. Probable diagnosis: 1, 2, 4. Possible diagnosis: 1, 2, 5.

**Table 2:** The tools for assessing sarcopenic dysphagia.

Evaluating Target	Tools
Muscle mass	Dual-energy X-ray absorptiometry (DXA), bioimpedance analysis (BIA)
Muscle strength	Dynamometer
Physical performance	Six-minute walk test (6MWT), Short Physical Performance Battery score (SPPB), five-time chair stand test (5TSTS), timed up-and-go test (TUG), 400 m walk test (400MWT)
Swallowing function	Eating Assessment Tool (EAT-10), dysphagia severity scale (DSS), repetitive saliva swallowing Test (RSST), Functional Oral Intake Scale (FOIS), Food Intake Level Scale (FILS), modified water swallowing test (MWST), videofluoroscopy swallowing study (VFSS)
Swallowing muscle strength	JMS tongue pressure measuring instrument (JMS, Hiroshima, Japan), Iowa Oral Performance Instrument (IOPI), jaw-opening force trainer KT2016 (Livet Inc., Tokyo, Japan), Lip de Cum (Cosmo Instruments Co., Ltd., Tokyo, Japan), surface electromyography(sEMG), high-resolution manometry (HRM)
Swallowing muscle mass	Ultrasonography, magnetic resonance imaging (MRI)

**Table 3:** The cut-off point of the tools for the diagnosis of sarcopenic dysphagia from the available literature.

Evaluating Tool	Cut-Off Point
**Muscle mass**	
**Dual-energy X-ray absorptiometry** **(DXA)**	<7.0 kg/m^2^ in men and <5.5 kg/m^2^ in women ^a^ <7.0 kg/m^2^ in men and <5.4 kg/m^2^ in women ^b^
**Bioimpedance analysis (BIA)**	<7.0 kg/m^2^ in men and <5.5 kg/m^2^ in women ^a^ <7.0 kg/m^2^ in men and <5.7 kg/m^2^ in women ^b^
**Muscle strength**	
**Dynamometer**	<27 kg for men and <16 kg for women ^a^ <28 kg for men and <18 kg for women ^b^
**Physical Performance**	
**6 min walk**	<0.8 m/s ^a^ <1.0 m/s ^b^
**Short Physical Performance Battery**	≤8 ^a^ ≤9 ^b^
**5-time chair stand test**	≥15 s ^a^ ≥12 s ^b^
**Timed up-and-go test**	≥20 s ^a^
**400 m walk**	≥6 min ^a^
**Swallowing function**	
**Eating Assessment Tool (EAT-10)**	≥3
**Dysphagia severity scale (DSS)**	≤4
**Repetitive saliva swallowing Test (RSST)**	≤2
**Functional Oral Intake Scale (FOIS)**	≤5
**Food Intake Level Scale (FILS)**	Not available
**Modified water swallowing test (MWST)**	Not available
**Videofluoroscopy swallowing study (VFSS)**	Not available
**Swallowing muscle strength**	
**Maximal isometric tongue pressure**	<20 kPa
**Jaw-opening force**	Not available
**Lip force**	<10.4 Newton for men and <8.5 Newton for women
**Surface electromyography (sEMG)**	<387.09% of jaw open contraction for maximal amplitude <1.96 s for total duration
**High-resolution manometry (HRM)**	Not available
**Swallowing muscle mass**	
**Ultrasonography**	<1536 mm^2^ for the cross-sectional area of the tongue muscle <75.1 mm^2^ for the cross-sectional area of the digastric muscle
**Magnetic resonance imaging (MRI)**	Not available

^a^ The criteria suggested by European Working Group on Sarcopenia in Older People (EWGSOP) in 2018; ^b^ the criteria suggested by Asian Working Group for Sarcopenia (AWGS) 2019 consensus.

## Data Availability

Data is contained within the article.
